# General Analytic Correction for Probe-Position Errors in Spherical Near-Field Measurements

**DOI:** 10.6028/jres.096.021

**Published:** 1991

**Authors:** L. A. Muth

**Affiliations:** National Institute of Standards and Technology, Boulder, CO 80303

**Keywords:** computer simulations, error correction, spherical far fields, spherical near fields

## Abstract

A general theoretical procedure is presented to remove known probe-position errors in spherical near-field data to obtain highly accurate far fields. We represent the measured data as a Taylor series in terms of the displacement errors and the ideal spectrum of the antenna. This representation is then assumed to be an actual near field on a regularly spaced error-free spherical grid. The ideal spectrum is given by an infinite series of an error operator acting on data containing errors of measurement. This error operator is the Taylor series without the zeroth-order term. The *n* th-order approximation to the ideal near field of the antenna can be explicitly constructed by inspection of the error operator. Computer simulations using periodic error functions show that we are dealing with a convergent series, and the error-correction technique is highly successful. This is demonstrated for a triply periodic function for errors in each of the spherical coordinates. Appropriate graphical representations of the error-contaminated, error-corrected and error-free near fields are presented to enhance understanding of the results. Corresponding error-contaminated and error-free far fields are also obtained.

## 1. Introduction

A recently developed analytic technique that can correct for probe-position errors in planar near-field measurements to arbitrary accuracy [1,2] is also applicable to spherical near-field data after appropriate modifications. The method has been used successfully to remove probe-position errors in the planar near field, leading to more accurate far-field patterns, even if the maximum error in the probe’s position is as large as 0.2 λ. Only the error-contaminated near-field measurements and an accurate probe-position error function are needed to be able to implement the correction. It is assumed that the probe-position error function is a characteristic of the near-field range and has been obtained using state-of-the-art laser positioning and precision optical systems. The method also requires the ability to obtain derivatives of the error-contaminated near field *defined* on an error-free regular grid with respect to the coordinates. In planar geometry the spatial derivatives are obtained using fast Fourier transforms (FFT) [1,2]; in spherical geometry the derivatives of Hankel functions for radial errors, and the derivatives of the spherical electric and magnetic vector basis functions for errors in the *θ* and *ϕ* coordinates are needed.

## 2. General Analytic Procedure

Let *b*(*x*) and $\hat{b}\left(x;\delta x\right)$ be the error-free and error-contaminated near fields at position *x*, and *δx* the probe-position error function. Here the position vector *x* can be given in planar (Cartesian), cylindrical, or spherical coordinates. Then,

$$\hat{b}\left(x;\delta x\right)=\left(1+T\right)b\left(x\right),$$ (1)

where *T* is a differential error operator. Since real measurements are taken on an *irregular* grid, *x + δx*, the measured values can be represented in terms of an *unknown* error-free near field *b*(*x*) and the Taylor series expansion of this field around the regular grid *x.* Thus, the error operator is nothing but the Taylor series operator without the leading zeroth-order term. The exact functional form of *T* depends on the coordinate system used in representing the near field. To solve for the error-free near field eq (1) can be inverted to yield

$$b\left(x\right)={\left(1+T\right)}^{-1}\hat{b}\left(x;\delta x\right),$$ (2)

which can then be expanded to any arbitrary order in *δx.* First, we expand eq (2) as

$$b\left(x\right)=\left(1-T+TT-TTT+TTTT-\ldots \right)\hat{b}\left(x;\delta x\right),$$ (3)

and observe that

$$T=t_{1}+t_{2}+t_{3}+t_{4}\ldots +t_{k}+\ldots .$$ (4)

In general, the *k*th-order term in the Taylor series

$$T\;\text{has}\;\text{the}\;\text{form}\;t_{k}=\frac{1}{k!}{\left(\delta s\right)}^{k}\frac{\partial^{k}}{\partial s^{k}},$$ (5)

where, in Cartesian coordinates, *s* is *x, y* or *z*, in cylindrical coordinates *s* is ρ, *ϕ* or *z*, and in spherical coordinates *s* is *r, θ*, or *ϕ.* Equation (4) can now be used to arrange the terms in eq (3) in an ascending order of approximation. Thus, to fourth-order in *δs*

$$\begin{array}{l} b\left(x\right)=(1\\ -t_{1}-t_{2}-t_{3}-t_{4}\\ +t_{1}t_{1}+t_{1}t_{2}+t_{1}t_{3}+t_{2}t_{1}+t_{2}t_{2}+t_{3}t_{1}\\ -t_{1}t_{1}t_{1}-t_{1}t_{2}t_{1}-t_{1}t_{1}t_{2}-t_{2}t_{1}t_{1}\\ +t_{1}t_{1}t_{1}t_{1})\hat{b}\left(x;\delta x\right). \end{array}$$ (6)

The explicit functional forms of *T, t_k_*, and eq (6) in Cartesian geometry (planar scanning) can be found in [1,2], where the question of convergence of the *n* th-order expansion has also been discussed.

The following observations about the structure of eq (6) are worthwhile:

- The first line of the equation is the *zeroth*-order approximation to the *ideal* near field and corresponds to the first term in eq (3).
- Each subsequent term in eq (3) gives rise to all the terms on a subsequent line in eq (6). For example, the term *TTT* gives rise to all the triple product terms in eq (6); all other terms originate from another term in eq (3).
- The sum of subscripts in each of the terms in eq (6) is 4 or less, indicating that we have written down a *fourth-order* approximation.
- All possible combinations of subscripts occur, subject to the constraint in (c).
- Fifth- or higher-order approximations can be quickly written down using observations (b), (c), and (d) as guidelines.

Finally, we make the following nontrivial observation: *b*(*x*) and $\hat{b}\left(x;\delta x\right)$ in eq (6) are both defined on a mathematically *regular* grid, even though originally the error-contaminated near field was obtained on an *irregular* grid. This shift in the definition of the error-contaminated field is an essential mathematical step in the error-correction procedure under consideration. The redefinition becomes important when *exact* derivatives of the error-contaminated near field on a *regularly* spaced grid are required; *by definition*, such derivatives can be obtained mathematically, but cannot be obtained experimentally. (In Cartesian geometry, or for planar near fields, derivatives can be obtained using Fourier techniques [1,2].)

The terms in eq (6) are differential operators acting on the error-contaminated near field *b*(*x; δx*). Terms such as *t*_1_*t*_3_ and *t*_3_*t*_1_ will yield different contributions as can be seen from the explicit expressions in Cartesian coordinates for probe-position errors in the *z* coordinate. Thus,

$$t_{1}t_{3}\hat{b}=\frac{1}{3!}\delta z\frac{\partial }{\partial z}{\left(\delta z\right)}^{3}\frac{\partial^{3}\hat{b}}{\partial z^{3}}$$ (7)

and

$$t_{3}t_{1}\hat{b}=\frac{1}{3!}{\left(\delta z\right)}^{3}\frac{\partial^{3}}{\partial z^{3}}\delta z\frac{\partial \hat{b}}{\partial z},$$ (8)

which show that different derivatives act on different functions in the two cases. Further, the derivatives of the error function *δz* as required by each of the terms in eq (6) cannot be measured and are only defined mathematically [1,2], subject to the constraint that each term satisfy Maxwell’s equations.

### 2.1 Simultaneous Errors in Two or More Coordinate Variables

The discussion so far has assumed that probe-position errors occur in only one coordinate variable at a time. In fact, simultaneous errors in more than one coordinate can be treated easily by generalizing eqs (4) and (6). This is accomplished by redefining *t_k_* in eq (5). We simply write

$$t_{1}=t_{1}^{\left(1\right)}+t_{1}^{\left(2\right)}+t_{1}^{\left(3\right)},$$ (9)

where the superscript indicates one of the three coordinates in use. These are just the three first-order terms that appear in the usual Taylor series expansion of any function of three variables. The definition of the second-order expression also needs to be augmented the same way, but additional terms must be included to account for the contribution from mixed derivatives. The general *t*_2_ term is now written as

$$t_{2}=\sum_{s}t_{2}^{\left(s\right)}+\sum_{s\neq s}t_{11}^{\left(s,s\prime \right)}$$ (10)

where $t_{11}^{\left(s,s\prime \right)}$ is

$$t_{11}^{\left(s,s\prime \right)}=\partial s\partial s\prime \frac{\partial^{2}}{\partial s\partial s\prime }.$$ (11)

Again these are just the second-order terms in the usual Taylor series expression. The definition of the third- and higher-order terms *t_k_* in eqs (4) and (6) can be generalized the same way, and when these general expressions are substituted into eq (6), we obtain the expression for the error-corrected near field in the presence of simultaneous errors in more than one coordinate. Obviously the number of terms in eq (6) quickly increases with the order of correction and with the number of error-contaminated coordinates considered.

## 3. Spherical Error Correction

In spherical scanning, near-field data are obtained on the surface of a sphere of radius *r*_0_ at regular *Δθ* and *Δϕ* intervals. The center of rotation is fixed and the probe points toward this center at every point of the spherical grid. At each point two measurements are taken, corresponding to the *θ* and *ϕ* components of the measured electric field.

To study the error-correction technique we will consider probe-position errors in a single spherical coordinate only. We also assume that the orientation of the probe is always correct, meaning that the probe points to the center of rotation independent of the position of the probe. To obtain error-correction expressions for errors in the *r, θ*, or *ϕ* coordinates the explicit form of *t_k_* has to be substituted into eqs (4) and (6). Thus, for errors in the radial coordinate, the *k*th-order Taylor series term is

$$t_{k}=\frac{1}{k!}{\left(\delta r\right)}^{k}\frac{\partial^{k}}{\partial r^{k}}.$$ (12)

Similar expressions can be immediately written down for errors in the *θ* and *ϕ* coordinates:

$$t_{k}=\frac{1}{k!}{\left(\delta \theta \right)}^{k}\frac{\partial^{k}}{\partial \theta^{k}}$$ (13)

and

$$t_{k}=\frac{1}{k!}{\left(\delta \phi \right)}^{k}\frac{\partial^{k}}{\partial \phi^{k}}.$$ (14)

These error functions depend on the coordinates: *δr* = *δr*(*r*_0_,*θ*,ϕ), *δθ* = *δθ*(*r*_0_,*θ*,ϕ), and *δϕ* = *δ*ϕ(*r*_0_,*θ,ϕ*) for fixed *r*_0_. This must be kept in mind when spherical versions of the expressions shown explicitly in eqs (7) and (8) are evaluated. When eqs (12), (13), and (14) are substituted into eq (6) we obtain the error-corrected spherical near field in terms of the error-contaminated (measured) spherical near field.

### 3.1 Spherical Near Fields

In spherical geometry, we really have two independent near fields, which are the *θ* and *ϕ* components of the electric field *E* measured by an ideal dipole. The tangential electric field *E_t_*, with wave-number *k*, can be expressed [3] in terms of an infinite sum of products of spherical Hankel functions of the first kind $h_{n}^{\left(1\right)}\left(kr\right)$ and spherical vector basis functions *X_nm_*(*θ,ϕ*). We can write (using $\hat{r}$ for the unit vector in the radial direction)

$$\begin{array}{c} E_{t}\left(r,\theta ,\phi \right)=\sum_{n}\sum_{m}[\alpha_{nm}^{\left(h\right)}h_{n}^{\left(1\right)}\left(kr\right)X_{nm}\left(\theta ,\phi \right)\\ +\alpha_{\mathrm{nm}}^{\left(e\right)}g_{n}^{\left(1\right)}\left(kr\right)\hat{r}\times X_{nm}\left(\theta ,\phi \right)], \end{array}$$ (15)

where, with *x* ≡ *kr*,

$$g_{n}^{\left(1\right)}\left(x\right)=\frac{1}{x}\frac{d}{dx}\left[xh^{\left(1\right)}\left(x\right)\right].$$ (16)

The near-field quantities *b* and $\hat{b}$ in eq (6) are identified with either the *θ* or *ϕ* component of *E*_t_ and *Ê_t_*, respectively, where *Ê_t_*, is the error-contaminated electric field. Only one set of electric coefficients $\alpha_{nm}^{\left(e\right)}$ and one set of magnetic coefficients $\alpha_{nm}^{\left(h\right)}$ appear in eq (15), and the error-correction procedure corrects both components of the measured spherical field simultaneously.

To obtain the coefficients *α_nm_*, in eq (15) we use the explicit definitions and orthogonality relations obeyed by the vector basis functions *X_nm_* [3]. These are $\sqrt{n\left(n+1\right)}X_{nm}\left(\theta ,\phi \right)=LY_{nm}\left(\theta ,\phi \right),$ where *L* = − *i*(*r* × ▽) is the the well-known angular momentum operator widely used in quantum mechanics, and the spherical harmonics *Y_nm_*(*θ*,*ϕ*) = *P_nm_*(*θ*) exp(*imϕ*), where *P_nm_* are associated Legendre functions. In component form,

$$\sqrt{n\left(n+1\right)}X_{nm}\left(\theta ,\phi \right)=-\left(m\frac{Y_{nm}}{\sin\theta },i\frac{\partial Y_{nm}}{\partial \theta }\right).$$ (17)

The orthogonality properties are [3]

$$\int_{0}^{2\pi }\int_{0}^{\pi }X_{nm}^{*}\cdot X_{n^{\prime }m^{\prime }}\sin\;\theta d\theta d\phi =\delta_{n^{\prime }n^{\prime }}\delta_{m^{\prime }m^{\prime }}$$ (18)

with a similar relationship for $\hat{r}\times X_{nm}^{*},$ and

$$\int_{0}^{2\pi }\int_{0}^{\pi }X_{n^{\prime }m^{\prime }}^{*}\cdot \hat{r}\times X_{nm}\sin\;\theta d\theta d\phi =0$$ (19)

for all *n, n′, m, m′*, where *δ_nm′_* is the Kronecker delta. The coefficients can now be obtained [3] using eqs (15), (18) and (19):

$$\alpha_{nm}^{\left(h\right)}h_{n}^{\left(1\right)}\left(kr\right)=\int_{ο}^{2\pi }\int_{ο}^{\pi }E_{t}\left(r,\theta ,\phi \right)\cdot X_{nm}^{*}\sin\theta d\theta d\phi$$ (20)

and

$$\begin{array}{l} \alpha_{nm}^{\left(e\right)}g_{n}^{\left(1\right)}\left(kr\right)=\\ \int_{0}^{2\pi }\int_{0}^{\pi }E_{t}\left(r,\theta ,\phi \right)\cdot \hat{r}\times X_{nm}^{*}\sin\;\theta d\theta d\phi . \end{array}$$ (21)

With eqs (18), (19), (20) and (21), any spherical near field, error-free or error-contaminated, can be cast into the form of eq (15), and given a set of coefficients *α_nm_*, a spherical vector function can always be constructed using eq (15). Consequently, each of the terms appearing in eq (6) and any factor *t_k_* in eq (6) can be evaluated in spherical coordinates. On a regular grid the summation can be accomplished using an efficient FFT summation, but on an irregular grid the sum must be evaluated directly, or by a Taylor series as described in [1,2].

To obtain the coefficients $\alpha_{nm}^{\left(h\right)}$ numerically we rewrite eq (20) as

$$\begin{array}{l} 2a_{nm}^{\left(h\right)}h_{n}^{\left(1\right)}\left(kr\right)=\\ 2\pi \int_{-\pi }^{\pi }{\tilde{E}}_{t}^{m}\left(r,\theta \right)\cdot {\tilde{X}}_{nm}^{*}\left(\theta \right)|\sin\theta |d\theta . \end{array}$$ (22)

Here the factor 2π is the result of the *ϕ* integral and the factor 2 on the left is introduced to offset the effect of extending the range of integration in *θ*. ${\tilde{E}}_{t}^{m}\left(r,\theta \right)$ and ${\tilde{X}}_{nm}\left(\theta \right)$ are the *ϕ*-transforms of *E_t_*(*r,θ,ϕ*) and *X_nm_*(*θ,ϕ*), respectively, extended into the range [−π,0]. The integrand in eq (22) is now an even function of *θ*, and can be expanded in a Fourier series,

$${\tilde{E}}_{t}^{m}\left(r,\theta \right)\cdot {\tilde{X}}_{nm}^{*}\left(\theta \right)|\sin\theta |=\sum_{l}c_{l}^{nm}e^{-il\theta }$$ (23)

where the coefficients *c_l_^nm^* can be obtained by Fourier transforming the data. Since only the coefficient $c_{ο}^{nm}$ will survive the term by term integration of the sum in eq (23), we immediately obtain from eqs (22) and (23)

$$\alpha_{nm}^{\left(h\right)}h_{t}^{\left(1\right)}\left(kr\right)=2\pi^{2}\cdot c_{ο}^{nm}.$$ (24)

Similar expressions can be written for $\alpha_{\mathrm{nm}}^{\left(e\right)}$ in eq (21).

### 3.2 Derivatives of Spherical Near Fields

To evaluate the terms and factors appearing in eq (6) in spherical coordinates, we must be able to obtain first- and higher-order derivatives of arbitrary spherical near fields with respect to any of the spherical coordinates. Derivatives with respect to *ϕ* are the simplest, since the *ϕ* dependence is only through the factors exp(*imϕ*) in the vector basis functions. Hence, a *k*th-order derivative with respect to *ϕ* will merely alter the coefficients in eq (15) according to the substitution,

$$\alpha_{nm}\to {\left(im\right)}^{k}\alpha_{nm},$$ (25)

after which the summation can be performed without change to the summation procedure in use. Radial derivatives are only somewhat more complicated; we obtain *k*th-order derivatives of Hankel functions with respect to *x* after repeated differentiation of the recursion relation [3],

$$\left(2n+1\right)\frac{dh_{n}^{\left(1\right)}\left(x\right)}{dx}=nh_{n-1}^{\left(1\right)}\left(x\right)-\left(n+1\right)h_{n+1}^{\left(1\right)}\left(x\right).$$ (26)

After Substitution of derivatives of Hankel functions in place of the functions themselves in eq (15), the existing summation procedure can be used without modification to obtain radial derivatives of the components of the near field. However, derivatives with respect to *θ* cannot be accomplished with ease, since no simple recursion relationship exists that can be utilized in a straighforward manner in a computer algorithm. To obtain *θ* derivatives we have to use Fourier series. If we assume that the *θ* dependence of the components (denoted by superscript *s*) of *E_t_* has been written in the form

$$\sum_{l}{c_{l}}^{\left(s\right)}e^{il\theta }$$ (27)

then the *k*th-order *θ* derivative is

$$\sum_{l}{\left(il\right)}^{k}{c_{l}}^{\left(s\right)}e^{il\theta }.$$ (28)

The coefficients *c_l_*^(s)^ can be obtained using a fast transform; after modification of the coefficients by the factors (*il*)*^k^*, the same FFT can be used to perform the summation indicated in eq (28). Since the data must be periodic with a period of 2π for analysis by an FFT, a near field defined on the *θ* interval [0,π] must be extended to the interval [0,2π] or [ − π,π] using the symmetry properties of the basis functions [4,5].

### 3.3 Data Analysis

As is evident from the discussion in the preceding sections, we need efficient numerical procedures for two basic computational problems arising from eq (15):

- Given a spherical vector function *E_t_*(*θ*,*ϕ*), we must be able to *analyze* it to obtain the coefficients *α_nm_*, and
- Given a set of coefficients *α_nm_*, we must be able to *synthesize* the spherical vector function *E_t_*(*θ*,*ϕ*) by performing the sum.

Focusing on a specific term of the full error-correction expression as given in eq (6), we can appreciate the role of these two computational procedures. We have, for the case of errors in the *r* coordinate,

$$t_{1}t_{2}\hat{b}=\frac{1}{2}\delta r\frac{\partial }{\partial r}{\left(\delta r\right)}^{2}\frac{\partial^{2}}{\partial r^{2}}\hat{b}\left(r,\theta ,\phi \right),$$ (29)

where $\hat{b}$ now stands for the components of *Ê_t_.* The following six steps must be executed to evaluate this expression numerically:

1. We analyze the components of *Ê_t_* to obtain coefficients *α_nm_*, as defined in eq (15).
2. We obtain the second-order radial derivatives of the components of *Ê_t_* by performing the summation in eq (15) using second-order derivatives of the radial functions.
3. We multiply the result by the function (*δr*)^2^, thereby obtaining a new spherical near field.
4. We analyze the fields obtained in step (3) to get a new set of coefficients *α_nm_*, as defined in eq (15).
5. We obtain the first-order radial derivatives of the components of *Ê_t_* by performing the summation in eq (15) using first-order derivatives of the radial functions.
6. We multiply the result in step (5) by 0.5 *δr* to obtain the part of the error-corrected spherical near field denoted by $t_{1}t_{2}\hat{b}$ in eq (6).

Similar sequences of steps will correctly evaluate any and all of the terms in eq (6) to obtain the ideal error-free near field. The procedure is highly recursive, and a few well designed subroutines can provide the result of the extensive and complex computational task called for in eq (6). The procedure is the same for errors in the *θ* and *ϕ* coordinates.

### 3.4 Computer Simulations

Computer simulations were performed for probe-position errors in a single spherical coordinate only; simultaneous errors in two or three coordinates were not considered. The following sequence of steps were performed for errors in each of the spherical coordinates:

1. We start with an error-free spherical near field and analyze it to obtain its expansion coefficients *α_nm_* [(see eq (15)].
2. We define a probe-position error function |*δ*x| = *δs*(*θ*,*ϕ*) to be studied, and choose its amplitude.
3. We construct an error-contaminated near field by performing the summation in eq (15) at the *irregular* grid points x + δx. This requires a direct sum at each point of the grid, since no efficient method of summing is known to exist on an irregular grid.
4. We perform the computations in eq (6) to obtain the error-corrected near field. The steps taken to accomplish this were outlined above in some detail (see eq (29) and the brief discussion following it).
5. We calculate error-free, error-contaminated, and error-corrected far fields.
6. We compare error-free, error-contaminated, and error-corrected fields to study the effectiveness of the error correction.

### 3.5 Results and Discussion

**Figure 1:**
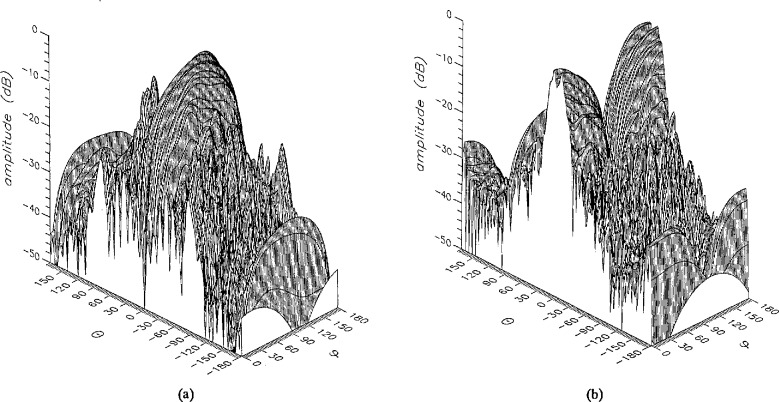
The amplitude of the error-free near field at 3.3 GHz, (a) the *θ* and (b) the *ϕ* component.

**Figure 2:**
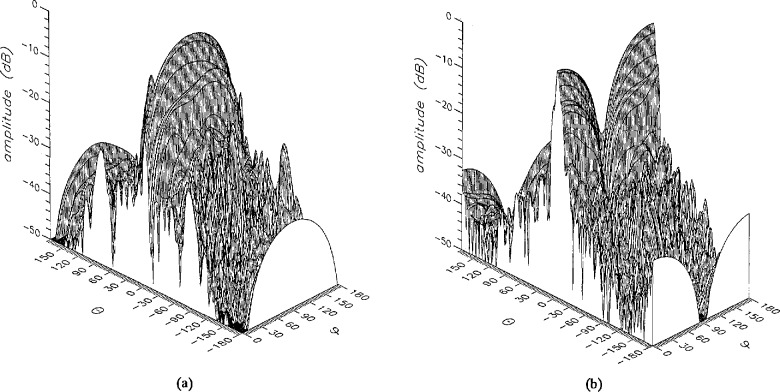
The amplitude of the error-free far field at 3.3 GHz, (a) the *θ* and (b) the *ϕ* component.

The near field used in all the simulations was generated by a microstrip array antenna consisting of four 16 × 16 element subpanels operating at 3.3 GHz. The scan radius was 128 cm. Figures 1a and 1b show perspective plots of the amplitudes of the *θ* and *ϕ* components of the error-free near field. The near field was obtained by summing the terms in eq (15) with *n* =30, after the original near-field data were analyzed to obtain coefficients up to *n* = 87. With *n* = 30, direct summations on irregular grids could be performed in about 6 hours on a personal computer. Figures 2a and 2b show perspective plots of the amplitudes of the *θ* and *ϕ* components of the error-free far field.

We chose periodic probe-position error functions of the form

$$\delta s\left(\theta ,\phi \right)=A\cos^{2}\alpha \theta \;\cos^{2}\beta \phi ,$$ (30)

where *s* = *r, θ*, or *ϕ*, and α = β = 3. For errors in the radial coordinate we chose *A* = 0.1 λ ≈ 1 cm, and for errors in the angular coordinates, we chose *A* = 0.01 λ, which corresponds to a maximum angular error of 3.6°. The magnitude of these errors are unrealistic, since, on the NIST spherical near field scanner, the probe’s position errors are estimated to be less than *δr* ≈ 0.1 cm, and *δθ* ≈ *δϕ* ≈ 0.5°. Periodic probe-position errors were chosen, because such errors in the near field could lead to large errors in the far field. This is a well known phenomenon in planar near-field to far-field transformations [6,7]. The procedure, however, could be easily performed with nonperiodic error functions.

The results of the simulations are presented in the figures 3–26: perspective plots of ratios of error-contaminated and error-free fields are presented for errors in the three coordinates separately, followed by perspective plots of ratios of error-corrected and error-free fields. Similar plots are presented for the far fields. Both amplitudes and phases are shown for all cases.

**Figure 3:**
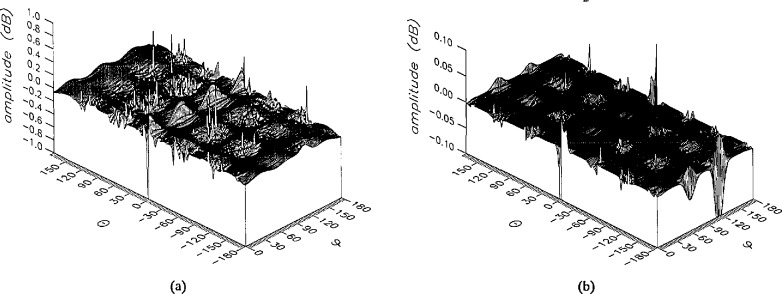
The amplitudes of the *θ* components of the ratios of (a) the error-contaminated and (b) the error-corrected near fields to the error-free near field in the case of errors in the *r* coordinate.

**Figure 4:**
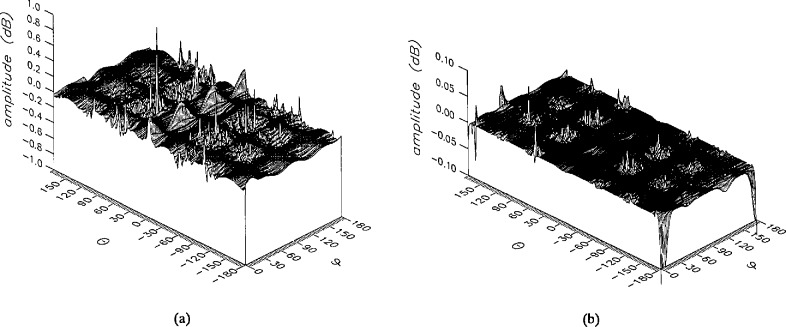
The amplitudes of the *ϕ* components of the ratios of (a) the error-contaminated and (b) the error-corrected near fields to the error-free near field in the case of errors in the *r* coordinate.

An examination of the plots immediately reveals the success of the error correction. By comparing the amplitudes of the error-contaminated and error-corrected ratios, we immediately observe insignificant levels of residual errors almost everywhere on the sphere. The same quantitative observation can be made about the phase difference plots, where the residual error in the error-corrected phases approaches 0. The following additional qualitative observations are worthwhile:

- The correction is most successful in the forward hemisphere, especially around the main beam at *θ* = 0 in all cases. This is true for both the near and the far field.
- The correction is least successful in the back hemisphere, especially around *θ* = *±* 180°, where the data are ill-determined and small in amplitude.
- There are no large regions on the sphere where the correction technique fails.
- At isolated points the correction seems to be less successful as evidenced by peaks in the perspective plots. These points correspond to deep nulls in the original error-free near field, and, consequently, can be understood as artifacts of the ratio field, rather than some more serious problem with the technique.
- The radial error function *δr*(*θ*,*ϕ*) clearly shows up in figures (3b) and (4b), as expected, since we have essentially plotted the phase of the ratio of Hankel functions of the form exp(*ikr*)/*r* at *r + δr*(*θ*,*ϕ*) and *r*, with *δr* ≤ *r*
- The three-lobe structure of the periodic error function over an angular interval of 180° in *θ* and ϕ shows up clearly in all the error-contaminated plots, as expected. This structure also shows up in the error-corrected plots, indicating that the error-correction procedure is a systematic global reduction of the error without altering the functional form of the error. This agrees with the structure of eq (6).

Both the qualitative and quantitative features of the results show that the error correction outlined in this study can be very useful in providing more accurate spherical near-field data to determine accurate far fields of antennas.

## 4. Suggestions for Further Study

Here we have demonstrated the effectiveness of a novel error-correction technique that removes probe-position errors in *r, θ*, or *ϕ* from spherical near-field data. For completeness, the technique should be applied when errors in all three coordinates are present simultaneously. This is the most realistic case. Such a *complete* error-correction technique would be computationally more complicated and extensive, but in principle not more difficult, and should also be effective and successful. Finally, more realistic probe-position error functions should be used, and the correction technique should be applied to real error-contaminated spherical data. The success of this error-correction technique is especially desirable at higher frequencies, where the realistic amplitudes of the probe-position errors on a spherical near-field range are a significant part of the wavelength.

## Figures and Tables

**Figure 5:**
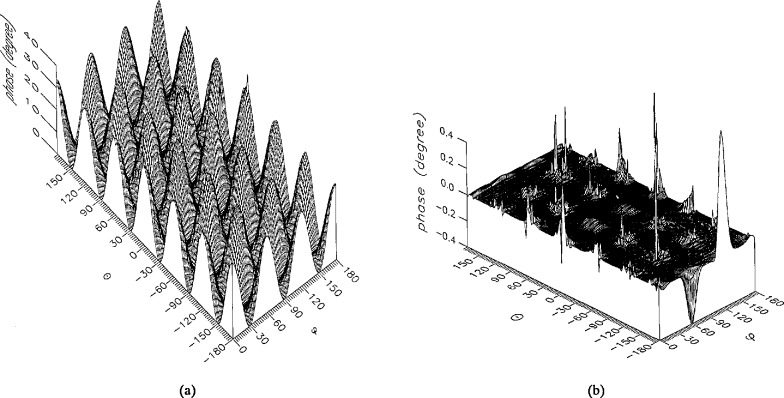
The phase of the *θ* components of the ratios of (a) the error-contaminated and (b) the error-corrected near fields to the error-free near field in the case of errors in the *r* coordinate.

**Figure 6:**
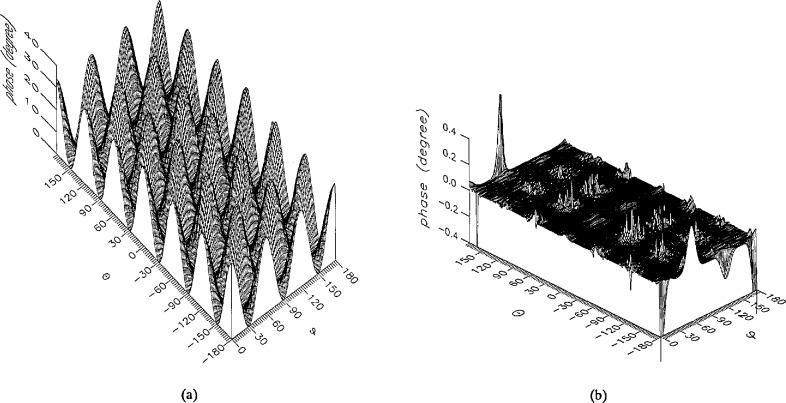
The phase of the *ϕ* components of the ratios of (a) the error-contaminated and (b) the error-corrected near fields to the error-free near field in the case of errors in the *r* coordinate.

**Figure 7:**
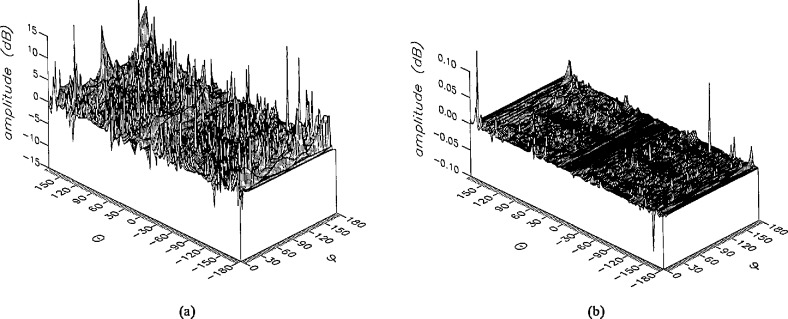
The amplitudes of the *θ* components of the ratios of (a) the error-contaminated and (b) the error-corrected far fields to the error-free far field in the case of errors in the *r* coordinate.

**Figure 8:**
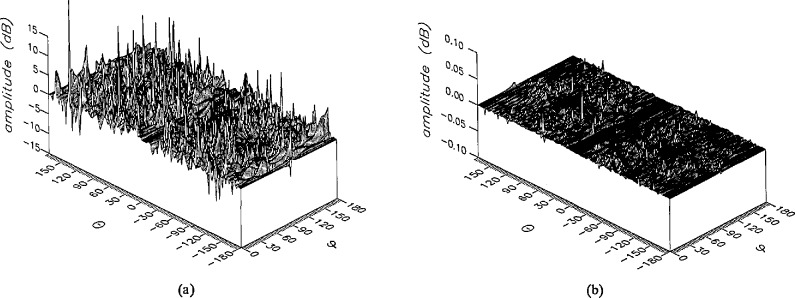
The amplitudes of the *ϕ* components of the ratios of (a) the error-contaminated and (b) the error-corrected far fields to the error-free far field in the ease of errors in the *r* coordinate.

**Figure 9:**
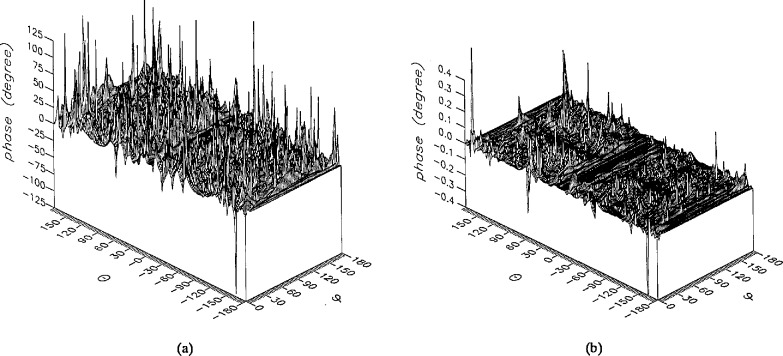
The phase of the *θ* components of the ratios of (a) the error-contaminated and (b) the error-corrected far fields to the error-free far field in the case of errors in the *r* coordinate.

**Figure 10:**
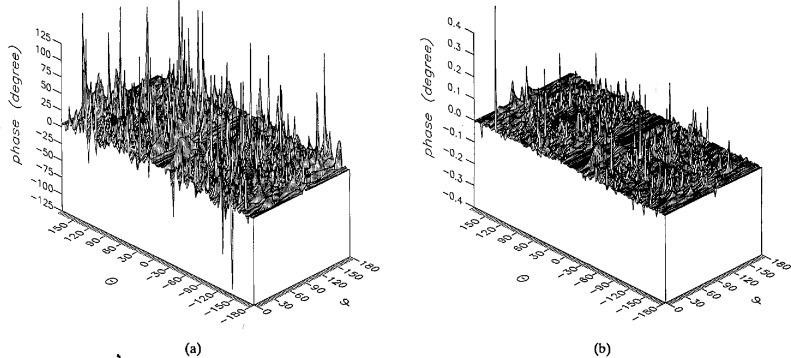
The phase of the *ϕ* components of the ratios of (a) the error-contaminated and (b) the error-corrected far fields to the error-free far field in the case of errors in the coordinate.

**Figure 11:**
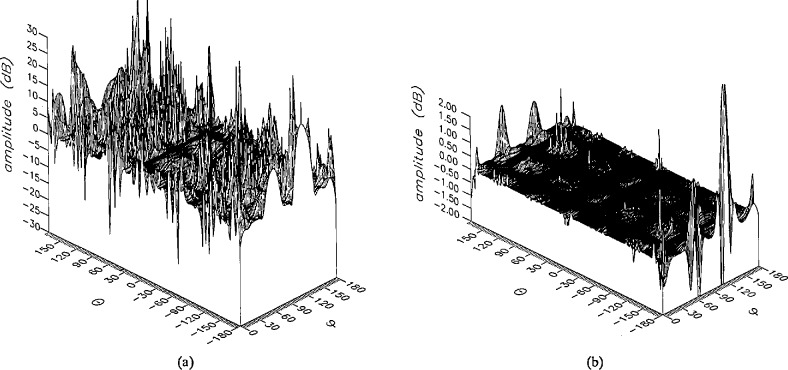
The amplitudes of the *θ* components of the ratios of (a) the error-contaminated and (b) the error-corrected near fields to the error-free near field in the ease of errors in the *θ* coordinate.

**Figure 12:**
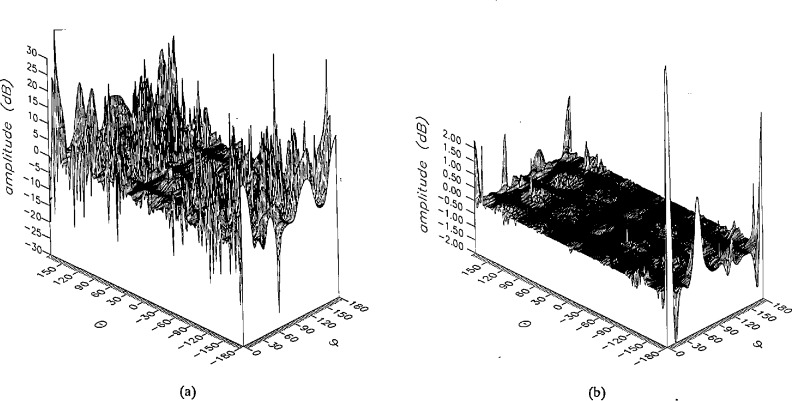
The amplitudes of the *ϕ* components of the ratios of (a) the error-contaminated and (b) the error-corrected near fields to the error-free near field in the case of errors in the *θ* coordinate.

**Figure 13:**
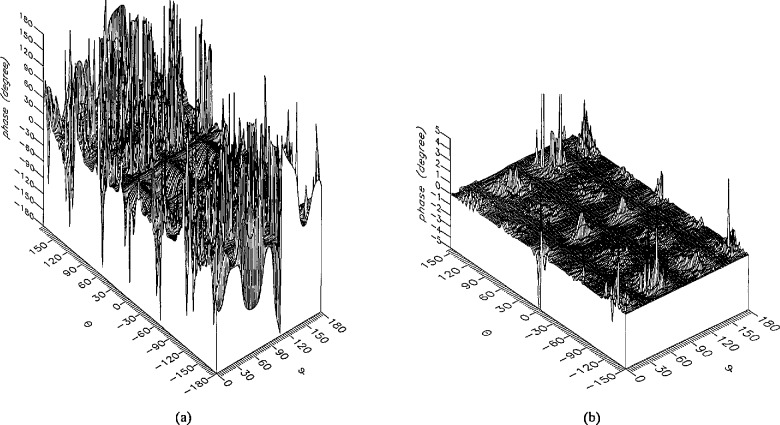
The phase of the *θ* components of the ratios of (a) the error-contaminated and (b) the error-corrected near fields to the error-free near field in the case of errors in the *θ* coordinate.

**Figure 14:**
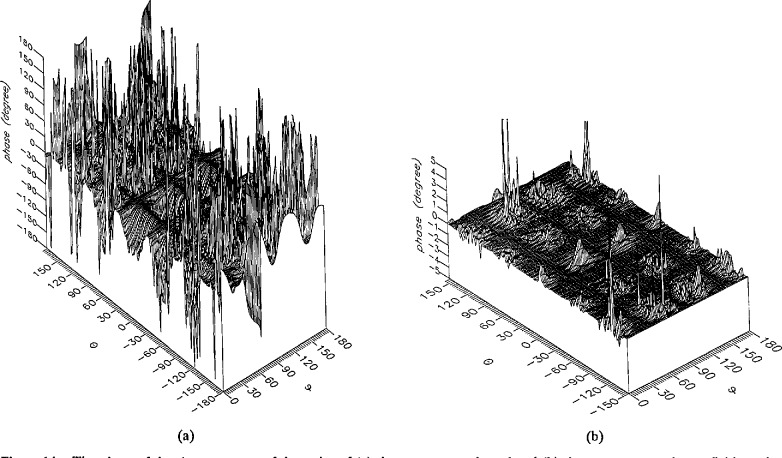
The phase of the *ϕ* components of the ratios of (a) the error-contaminated and (b) the error-corrected near fields to the error-free near field in the case of errors in the *θ* coordinate.

**Figure 15:**
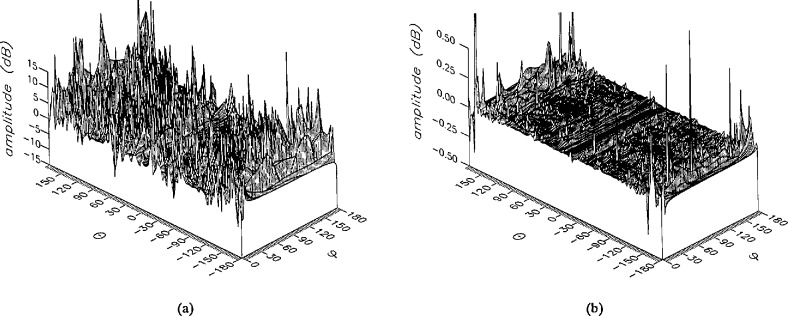
The amplitudes of the *θ* components of the ratios of (a) the error-contaminated and (b) the error-corrected far fields to the error-free far field in the case of errors in the *θ* coordinate.

**Figure 16:**
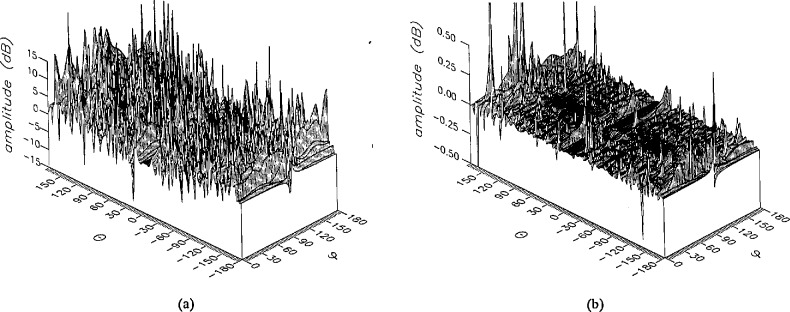
The amplitudes of the *ϕ* components of the ratios of (a) the error-contaminated and (b) the error-corrected far fields to the error-free far field in the case of errors in the *θ* coordinate.

**Figure 17:**
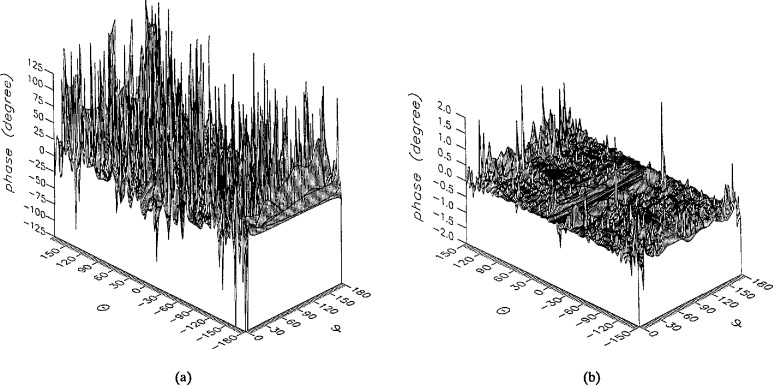
The phase of the *θ* components of the ratios of (a) the error-contaminated and (b) the error-corrected far fields to the error-free far field in the case of errors in the *θ* coordinate.

**Figure 18:**
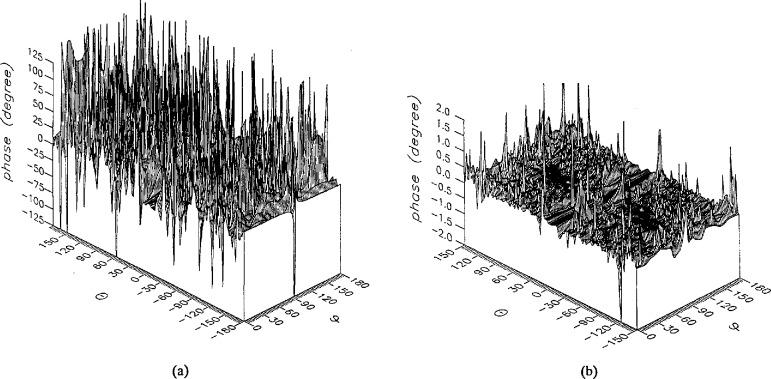
The phase of the *ϕ* components of the ratios of (a) the error-contaminated and (b) the error-corrected far fields to the error-free far field in the case of errors in the *θ* coordinate.

**Figure 19:**
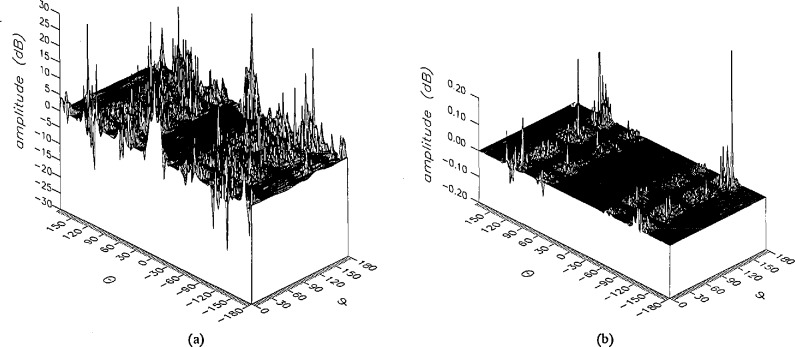
The amplitudes of the *θ* components of the ratios of (a) the error-contaminated and (b) the error-corrected near fields to the error-free near field in the case of errors in the *ϕ* coordinate.

**Figure 20:**
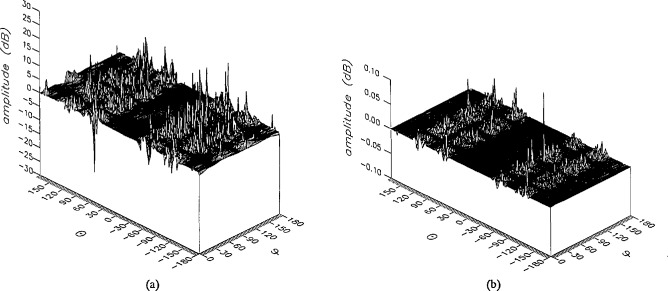
The amplitudes of the *ϕ* components of the ratios of (a) the error-contaminated and (b) the error-corrected near fields to the error-free near field in the case of errors in the *ϕ* coordinate.

**Figure 21:**
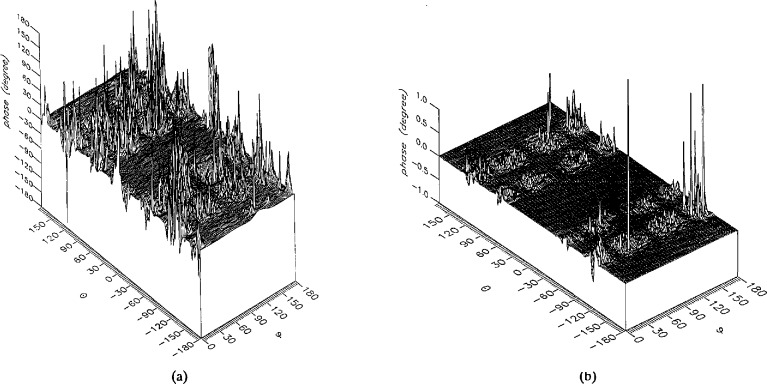
The phase of the *θ* components of the ratios of (a) the error-contaminated and (b) the error-corrected near fields to the error-free near field in the ease of errors in the *ϕ* coordinate.

**Figure 22:**
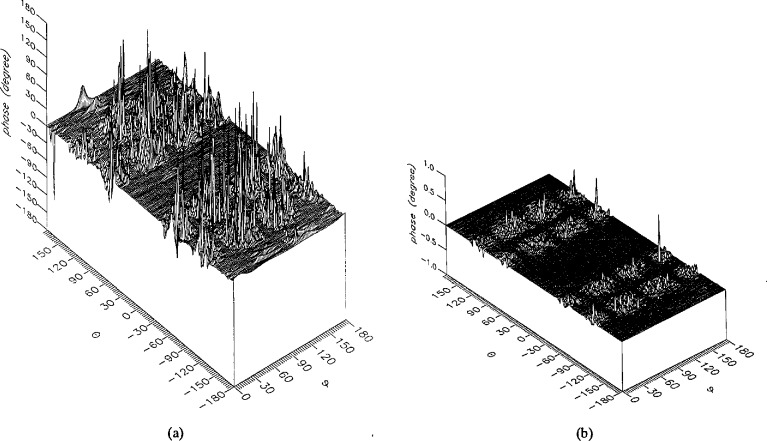
The phase of the *ϕ* components of the ratios of (a) the error-contaminated and (b) the error-corrected near fields to the error-free near field in the case of errors in the *ϕ* coordinate.

**Figure 23:**
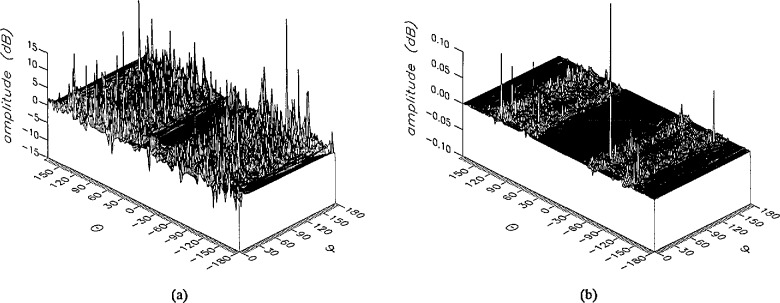
The amplitudes of the *θ* components of the ratios of (a) the error-contaminated and (b) the error-corrected far fields to the error-free far field in the case of errors in the *ϕ* coordinate.

**Figure 24:**
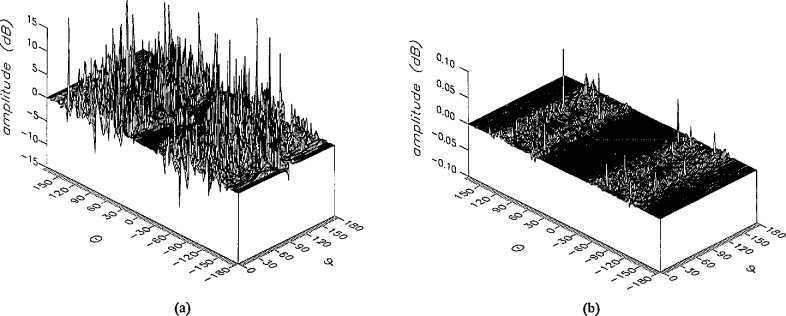
The amplitudes of the *ϕ* components of the ratios of (a) the error-contaminated and (b) the error-corrected far fields to the error-free far field in the case of errors in the *ϕ* coordinate.

**Figure 25:**
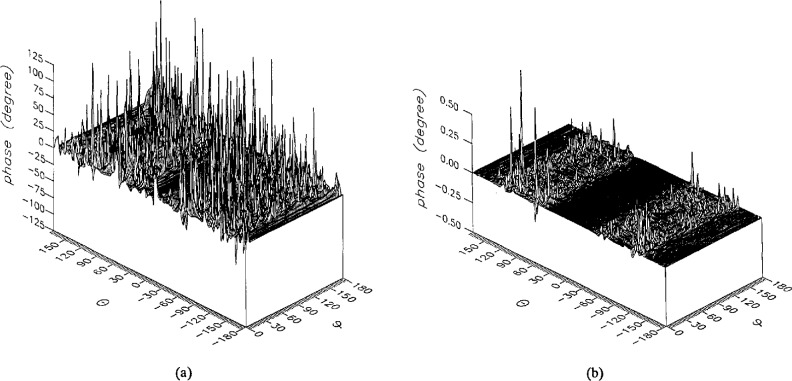
The phase of the *θ* components of the ratios of (a) the error-contaminated and (b) the error-corrected far fields to the error-free far field in the case of errors in the ϕ coordinate.

**Figure 26:**
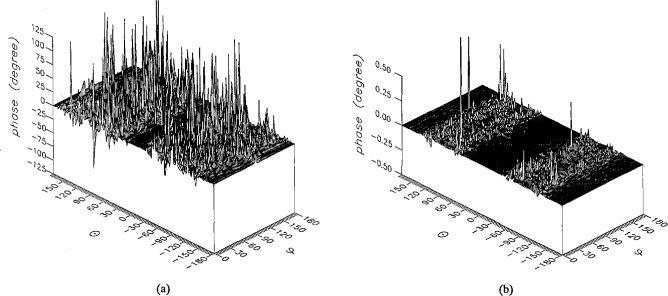
The phase of the *ϕ* components of the ratios of (a) the error-contaminated and (b) the error-corrected far fields to the error-free far field in the case of errors in the *ϕ* coordinate.
